# Convergence Insufficiency, Accommodative Insufficiency, Visual Symptoms, and Astigmatism in Tohono O'odham Students

**DOI:** 10.1155/2016/6963976

**Published:** 2016-07-20

**Authors:** Amy L. Davis, Erin M. Harvey, J. Daniel Twelker, Joseph M. Miller, Tina Leonard-Green, Irene Campus

**Affiliations:** ^1^Department of Ophthalmology and Vision Science, The University of Arizona, Tucson, AZ 85711, USA; ^2^Mel and Enid Zuckerman College of Public Health, The University of Arizona, Tucson, AZ 85724, USA; ^3^College of Optical Sciences, The University of Arizona, Tucson, AZ 85721, USA

## Abstract

*Purpose*. To determine rate of convergence insufficiency (CI) and accommodative insufficiency (AI) and assess the relation between CI, AI, visual symptoms, and astigmatism in school-age children.* Methods*. 3rd–8th-grade students completed the Convergence Insufficiency Symptom Survey (CISS) and binocular vision testing with correction if prescribed. Students were categorized by astigmatism magnitude (no/low: <1.00 D, moderate: 1.00 D to <3.00 D, and high: ≥3.00 D), presence/absence of clinical signs of CI and AI, and presence of symptoms. Analyses determine rate of clinical CI and AI and symptomatic CI and AI and assessed the relation between CI, AI, visual symptoms, and astigmatism.* Results*. In the sample of 484 students (11.67 ± 1.81 years of age), rate of symptomatic CI was 6.2% and symptomatic AI 18.2%. AI was more common in students with CI than without CI. Students with AI only (*p* = 0.02) and with CI and AI (*p* = 0.001) had higher symptom scores than students with neither CI nor AI. Moderate and high astigmats were not at increased risk for CI or AI.* Conclusions*. With-the-rule astigmats are not at increased risk for CI or AI. High comorbidity rates of CI and AI and higher symptoms scores with AI suggest that research is needed to determine symptomatology specific to CI.

## 1. Introduction

Convergence insufficiency (CI) is defined by the inability to accurately converge, or sustain accurate convergence, at near. Accommodative insufficiency (AI) is demonstrated by an insufficient amplitude of accommodation relative to age-based expectations. There is a high rate of comorbidity of CI and AI [1]. Convergence (C) and accommodation (A) are linked: when one accommodates for near focus, the eyes converge (as quantified by the AC/A ratio) and when one converges, the eyes accommodate (as quantified by the C/AC ratio) [1, 2]. Symptoms of CI can include significant asthenopia during near tasks: headache, diplopia, words appearing to move or jump, lack of concentration, visual fatigue, reading problems, blurred vision, and sore eyes [3]. AI has similar symptomatology: blurred vision, headache, and visual discomfort or fatigue [3].

The prevalence of CI and AI in the general population is not known due to an absence of population based epidemiological studies [4, 5]. School-based samples can provide a useful estimate of the potential impact of CI and AI in school-age children. However, estimates from currently available school-based samples are complicated by use of different exclusionary criteria (often making samples nonrepresentative of the overall school population), measurement methods, and diagnostic criteria across studies [1, 6–9]. In a study of Canadian school children, Letourneau and Ducic reported a 2.3% frequency of CI, with CI defined as receded near point convergence (NPC) and exophoria greater at near than at distance [6]. The literature also includes three studies of school children in the United States (California) that used similar methodology and diagnostic criteria. Criteria for high suspect/definite CI were presence of exophoria at near greater than at distance in addition to insufficient positive fusional vergence (PFV) and/or receded NPC. The criterion for AI was 2D below Hofstetter's minimum age expected accommodative amplitude (AA) [10]. Rouse et al. reported a CI rate of 13.0% (4.9% CI in the absence of AI) [7]. Borsting et al. reported CI in 17.3% of their sample (10.5% CI in the absence of AI) and AI in 17.3% (10.5% AI in the absence of CI) [8]. Marran et al. reported CI in 18.0% of their sample (14.7% CI in the absence of AI) and AI in 8.0% (4.7% AI without CI) [1]. More recently, Wajuihian and Hansraj assessed CI using the same criteria in a random sample of high school students in South Africa and reported CI in 12.2% of their sample (10.2% CI without AI) and AI in 4.5% (2.6% AI without CI); AI was defined by reduced accommodative amplitude combined with high values on monocular estimation retinoscopy and/or poor accommodative facility [9]. These studies of school-based samples have reported CI rates ranging from 2.3% to 18.0% when using presence of at least 2 clinical signs to define CI (4.9% to 10.3% CI without AI) and AI rates ranging from 4.5% (2 clinical signs) to 17.3% (1 clinical sign) (2.6% to 10.5% AI without CI). However, there are some limitations to these studies. Although Letourneau's study included the greatest sample size, near point of convergence was assessed utilizing a nonaccommodative target (penlight), in contrast to the other studies which used letter targets [1, 6–9]. Some studies excluded children with poor visual acuity and refractive error, thereby limiting generalizability of the reports and extent to which the samples represent the school population [1, 7, 8]. Other studies did not exclude children based on refractive error but also did not include information on number of children with refractive error who wore their correction for testing [6, 9]. The management of refractive error is significant with respect to assessing frequency of CI and AI. We previously reported that uncorrected astigmatic students showed difficulty in stimulating an accurate accommodative response [11]. However, Cacho-Martínez et al. observed that although accommodative dysfunction was associated with increased visual symptoms in a sample of university students, the effect is eliminated when analyses adjusted for uncorrected refractive error [12]. In addition, Dwyer and Wick observed that vergence or accommodative anomalies present in patients with uncorrected ametropia can often be alleviated after a period of spectacle wear [13]. This effect was most prominent in astigmatic patients, with greater recovery from vergence anomalies after correction of against-the-rule (ATR) astigmatism (67%) than with-the-rule (WTR) astigmatism (45%) of the patients. Finally, all of these studies focused on clinical criteria for CI and AI. Not all students with clinical signs of CI or AI are symptomatic however, and therefore these frequency estimates may not accurately represent the percentage of students requiring intervention.

The aims of the present study are to determine the frequency of convergence insufficiency (CI) and accommodative insufficiency (AI), assess the relation between CI, AI, and visual symptoms, and assess the relation between AI, CI, and astigmatism magnitude in a school-age sample. This report is unique in that we (a) report on frequency of CI and AI in a school-based (rather than clinic/patient-based) sample, (b) include students with significant refractive error so that our sample is more representative of the school population, (c) conduct testing with significant refractive errors corrected so that uncorrected refractive error does not result in overestimation of CI and AI, (d) report on CI and AI rates based on clinical measurements only (clinical CI and AI) and based on both clinical and symptom measurements (symptomatic CI and AI), and (e) assess the relation between refractive error (specifically astigmatism) and rate of CI and AI.

## 2. Methods

*Subjects*. Participants were third- through eighth-grade students who attended school on the Tohono O'odham Reservation during the 2013/2014 school year. All students in the targeted grades were eligible to participate. The Tohono O'odham, a Native American Tribe whose reservation is located in the Southwestern United States, have a high prevalence of with-the-rule (WTR) astigmatism [14].

This research followed the tenets of the Declaration of Helsinki and was approved by the Tohono O'odham Nation and the University of Arizona Institutional Review Board. This study was conducted in a manner compliant with the Health Insurance Portability and Accountability Act. Parents provided written informed consent and students provided written assent prior to participation.

*Procedures*. A complete cycloplegic eye examination was performed on each participant. Autorefraction utilizing the Retinomax K-Plus 2 Autorefractor (Nikon, Inc., Melville, NY) and subjective refinement were conducted at least 30 minutes after the administration of the following three drops: 0.5% proparacaine, 1% tropicamide, and 1% cyclopentolate. A spectacle correction was prescribed for students with significant refractive error (astigmatism ≥ 1.00 D in either eye, myopia: ≥ 0.75 D on any meridian in either eye, hyperopia: ≥ 2.50 D on any meridian in either eye, and anisometropia ≥ 1.50 D spherical equivalent (SEQ)). For astigmatism and myopia, the full correction was prescribed. For hyperopia, the spherical correction was reduced symmetrically by 1/3 or by 1.00 D, whichever was greater.

Binocular vision testing was performed on a second test day (after the eye examination). Students who wear prescribed spectacles were tested after a spectacle adaptation period of at least two weeks and were tested while wearing their spectacles. Binocular vision testing was conducted using the Convergence Insufficiency Treatment Trial (CITT) measurement protocols [15]. Testing included cover testing at distance and near, near point of convergence (NPC), both positive fusional vergence (PFV) and negative fusional vergence (NFV) at near, monocular AA measurement, and completion of the Convergence Insufficiency Symptom Survey (CISS) [16]. Cover testing was performed both at distance with isolated 20/30 letters at 6 m and at near with isolated 20/30 letters at 40 cm. NPC was measured three times as the distance where the student reported sustained blur or an objective observation of loss of fusion using the Astron International (ACR/2) accommodative rule (Gulden Ophthalmics, Elkins Park, PA) and printed Gulden fixation target (the equivalent of 20/30 at 40 cm), with the mean value used in analyses. NFV and PFV testing was performed three times at 40 cm using a horizontal prism bar utilizing a single column of letters of 20/30 equivalent as a fixation target, with the mean value used in analyses. Donder's push-up method of measuring monocular AA was performed once; the left eye was occluded and then utilizing the Astron Accommodative Rule (a printed Gulden fixation target consisting of a column of 20/30 letters at 40 cm) the distance at which the student reported the first sustained blur was noted. The CISS was administered by the examining doctor and scored using the standard method.

*Data Analysis*. Students with a constant heterotropia or any other ocular abnormalities other than refractive error were excluded from analyses. Students were classified as having no/low (<1.00 D), moderate (≥1 D to <3 D), or high astigmatism (≥3 D) based on magnitude of astigmatism in the most astigmatic eye (measured by cycloplegic refraction).

*Comparison with Previous School-Based Samples*. In order to compare our results with other studies, we first categorized students based on whether or not the following clinical signs were present:

1. an exodeviation at near at least 4 Δ greater than at far,
2. a receded NPC break (6 cm or greater),
3. insufficient PFV at 40 cm (i.e., failing Sheard's criterion (PFV less than twice the near phoria) or minimum PFV of ≤ 15 Δ base-out blur or break).

Students having clinical sign #1 in addition to signs #2 or #3 met criteria for having “common” CI (2 signs) and students having all three signs were classified as having “classic” or clinical CI [5]. Students were classified as having AI if they had accommodative amplitude (AA) at least 2 diopters below minimum age-based norms as defined by Hofstetter's formula (15 − 0.25 [age]) measured utilizing Donder's push-up method.

*Primary Analyses*. For the purpose of the present study, students meeting all three clinical criteria (above) are referred to as having “clinical CI” (also referred to as “classic” CI [5]). The subgroup of these students who met the criteria for “clinical CI” and had a CISS score ≥ 16 were classified as having “symptomatic CI.” Students were classified as having “clinical AI” if they had accommodative amplitude (AA) at least 2 diopters below minimum age-based norms as defined by Hofstetter's formula (15 − 0.25 [age]). Students meeting clinical AI criteria and having a CISS score ≥ 16 were classified as having “symptomatic AI.”

*Data Analysis*. Chi-square (*χ* ^2^) analysis was used to assess the relation between clinical CI and clinical AI and between symptomatic CI and symptomatic AI. A one-way Analysis of Variance (ANOVA) was conducted to compare mean CISS score across students divided into the following groups: did not meet the criteria for clinical CI or AI (neither CI or AI), met the criteria for clinical CI but not for AI (CI only), met the criterion for clinical AI but not for CI (AI only), and met the criteria for both clinical CI and AI. *χ* ^2^ was used to compare the rates of CI and AI by astigmatism magnitude (no/low, moderate, and high astigmatism).

## 3. Results

Of the 495 students who completed the eye examination and binocular vision testing, 11 were excluded from analyses due to presence of exotropia (*n* = 5), esotropia (*n* = 3), history of strabismus surgery (*n* = 1), or other abnormalities (asymmetric pupils, marked retinal and refractive changes). The final sample included 484 students (51% female), with average age 11.67 years (SD 1.81, range 8.26 to 15.87), 43.8% (212) with no/low astigmatism, 26.0% (126) with moderate astigmatism, and 30.2% (146) with high astigmatism in the most astigmatic eye.

*Relation between CI and AI*.  Table 1 shows the rate of common or classic/clinical CI (having 2 or 3 clinical signs) and clinical AI in comparison to other school-based samples.  Table 2 assesses the relation between clinical CI (3 clinical signs) and clinical AI. AI was present in 55.6% of students with clinical CI, but in only 29.5% of students who did not meet the CI criteria (*χ* ^2^ = 14.82, *p* < 0.001).  Table 3 assesses the relation between symptomatic CI and symptomatic AI. Symptomatic AI was present in 56.7% of students with symptomatic CI, but in only 15.6% of students who did not meet the symptomatic CI criteria (*χ* ^2^ = 31.84, *p* < 0.001). In both comparisons, the rate of AI was significantly higher in children with CI.

**Figure 1:**
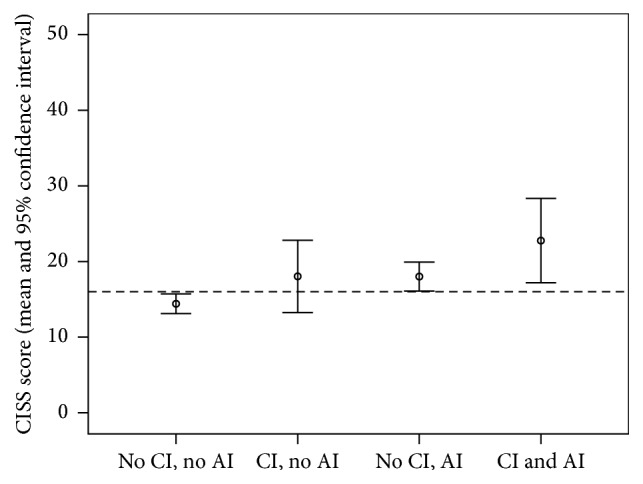
Mean Convergence Insufficiency Symptom Survey (CISS) score by presence/absence of clinical convergence insufficiency (CI) and clinical accommodative insufficiency (AI). Dashed line represents the CITT cutoff score for “symptomatic” CI.

*CI, AI, and Visual Symptoms*. A one-way ANOVA was conducted to compare mean CISS score across students divided into the following groups: did not meet the criteria for clinical CI or AI (neither CI nor AI, mean 14.42, SD 11.45), met the criteria for clinical CI but not for AI (CI only, mean 18.04, SD 11.32), met the criterion for clinical AI but not for CI (AI only, mean 18.02, SD 10.94), and met the criteria for both clinical CI and AI (mean 22.77, SD 14.95). Results indicated that students with AI only and students with both CI and AI had significantly higher mean CISS scores than students with neither CI nor AI (see Figure 1). Students with CI only did not have significantly elevated CISS scores on average, compared to students with neither CI nor AI.

*Relation between CI and Astigmatism and AI and Astigmatism*.  Table 4 shows the relation between presence of CI and AI (clinical and symptomatic) and astigmatism magnitude compared to a comparison group of students who did not meet the clinical criteria for either CI or AI (in italic). No significant differences were observed (all *p* values > 0.10).

## 4. Discussion

The present study reports frequency of CI and AI in a Tohono O'odham school-based sample. This study is unique in that we included students with significant refractive error and conducted testing with significant refractive errors that were corrected after a spectacle adaptation period. This is an important aspect of our design, as it has been reported that correction of ametropia results in the resolution of many vergence and accommodative conditions [13], and therefore correction of refractive error and spectacle adaptation period are likely to yield a more accurate assessment of the true prevalence of vergence anomalies. Additional unique aspects of this study are the reporting of frequency of* symptomatic* CI and AI and of the assessment of the relation between astigmatism and CI and AI.

The data in Table 1 indicates that frequency rates for clinical signs of CI and AI observed in Tohono O'odham children are higher than studies of other school-age samples when similar clinical criteria are applied. It is possible that this difference across studies accurately reflects a higher rate of clinical signs of CI and AI in this Tohono O'odham student population. However, it is also possible that some of these differences across studies are due to methodological differences, as previous studies excluded students with significant refractive error and/or poor acuity [1, 8], excluded students with significant refractive error if they were not currently wearing correction [7], or did include students with refractive error but did not report the number of children with refractive error tested with or without correction [6, 9].

When a more strict clinical diagnostic criteria for CI is used (i.e., presence of 3, rather than either 2 or 3, clinical signs) frequency rates are much lower (Table 2). When presence of symptoms is included in diagnostic criteria (Table 3), frequency of symptomatic CI was 6.2% (2.7% without AI) and symptomatic AI 18.2% (14.7% without CI). Other reports on school-based samples have not included symptom severity in their diagnostic criteria, so it is not clear how our findings compare to other school-based populations. However, as shown in Table 1, clinical signs of CI and AI appear to be more common in Tohono O'odham children, and therefore it is likely that symptomatic CI and AI would also be elevated in comparison to other populations. It is possible that our rates of symptomatic CI and AI are overestimated because although all students in the targeted grades were eligible to participate, symptomatic students may have been more likely to enroll in the study. In addition, AI results should be interpreted with caution as the classification of AI was made utilizing a single measure, whereas NPC and PFV for assessment of CI were measured 3 times (with the mean used in analyses).

Our findings confirmed previous reports indicating high comorbidity of CI and AI (Tables 2 and 3). In addition, we observed that students with clinical AI only and students with both clinical CI and AI had higher mean CISS scores than students with neither CI nor AI clinical signs whereas mean CISS scores for students with CI only did not significantly differ from students with neither CI nor AI. This pattern of results is very similar to results reported by Marran et al.: children with AI only and children with both AI and CI had elevated CISS symptom scores compared to children with normal binocular vision, and children with CI only had symptom levels similar to children with normal binocular vision [1]. Our findings lend further support to Marran et al.'s conclusion that elevated symptom scores in CI may be the result of comorbid AI.

As previously noted, rates of clinical signs of CI and AI in our sample were higher than rates reported in other school-based samples (Table 1). In our final analysis, we assessed the possibility that the higher rate of clinical signs may be associated with the high prevalence of with-the-rule astigmatism in Tohono O'odham children. Difficulty in stimulating an accurate accommodative response has been noted in high astigmats [11]; difficulty in stimulating an accommodative convergence response could increase the risk for CI. If a patient cannot accommodate properly, the visual target is blurry and accommodative convergence is not stimulated accurately, perhaps resulting in an increased risk of CI or AI. However, this hypothesis was not supported by the data: students with moderate to high astigmatism were not at an increased risk for CI or AI when wearing their best correction. We previously reported that uncorrected astigmatic students from this population showed difficulty in stimulating an accurate accommodative response [11]; the absence of elevated rates of CI and AI in astigmatic students indicates that either this accommodative difficulty did not lead to an increase of the risk for CI or, more likely, the spectacle correction of astigmatism alleviated clinical signs of CI or AI. However, students were not tested without their correction, so we cannot say definitively that spectacle correction did or did not reduce symptoms of CI or AI that may have been present when uncorrected. Our study is the first to report on the relation between astigmatism and CI and AI. However, all astigmatic students in this study had WTR astigmatism and therefore it is not clear if these results can be generalized to students with against-the-rule or oblique astigmatism.

In summary, we observed that Tohono O'odham children appear to have higher rates of clinical characteristics associated with CI and AI (Table 1). It is not clear if this finding is due to methodological differences across studies or if there is simply a higher rate of CI and AI in this population of students. However, our results indicate the elevated rates of CI and AI are not related to the high prevalence of astigmatism in this population, as corrected astigmatic children demonstrated no greater risk of CI or AI, compared to their nonastigmatic cohorts (Table 4). The current study and most previous studies reporting rates of CI and AI in school-based samples did not use sampling methods that allow for prevalence estimates representative of the overall population. As a result, further research on the prevalence of CI and AI is needed. We recommend that future studies report refractive error and spectacle wear so that the influence of uncorrected refractive errors can be considered in interpretation of results. Additional research is needed to further determine the symptomatology specific to CI due to the high comorbidity of CI and AI and the finding that elevated symptom scores were observed in students with AI (with or without CI), but not in students with CI only. Finally, it would be beneficial for further research into the prevalence of CI and AI to report the rates of* symptomatic* CI and AI, as these children are most likely to benefit from treatment.

## Figures and Tables

**Table 1:** Rate of convergence insufficiency (“common” (2 clinical signs) or “classic”/clinical (3 clinical signs)) and accommodative insufficiency (AI) in school-based study samples.

Study	Age in years: range Mean (SD)		*N*	CI^*∗*^	CI^*∗*^ only, no AI^†^	AI^†^	AI^†^ only, no CI^*∗*^	CI^*∗*^ and AI^†^
Letourneau and Ducic [6]	6 to 13 —		1954	2.3%	—	—	—	—

Rouse et al. [7]	9 to 13 11.3 (0.6)		453	13.0%	4.9%	—	—	—

Borsting et al. [8]	8 to 15 10.46 (1.41)		392	17.3%	10.5%	17.3%	10.5%	6.9%

Marran et al. [1]	— 11.5 (0.63)		299	18.1%	14.7%	8.0%	4.7%	3.3%

Wajuihian and Hansraj [9]	13 to 19 16.27 (1.79)		1201	12.2%	10.3%	4.5%	2.6%	1.9%

Present study	8 to 15 11.67 (1.81)	All students	484	31.4%	16.7%	32.4%	17.8%	14.7%
		No/low astigmati*s*m	212	26.9%	11.8%	33.0%	17.9%	15.1%
		Moderate astigmatism	126	34.1%	22.2%	31.0%	19.0%	11.9%
		High astigmatism	146	35.6%	19.2%	32.9%	16.4%	16.4%

^*∗*^Convergence insufficiency (CI): presence of 2 or 3 clinical signs (exophoria at near greater than at far in addition to insufficient PFV and/or receded NPC) for all studies except Letourneau and Ducic [6] (defined only by near point of convergence >10 cm and exophoria greater at near than at distance).

^†^Accommodative insufficiency (AI): accommodative amplitude (AA) 2D from Hofstetter's minimum age expected AA, except for Wajuihian and Hansraj who defined AI by reduced accommodative amplitude combined with high values on monocular estimation retinoscopy and/or poor accommodative facility.

**Table 2:** Relation between clinical convergence insufficiency (3 clinical signs present) and clinical accommodative insufficiency.

Clinical convergence insufficiency (CI)	Clinical accommodative insufficiency (AI)		Total
	No	Yes	
No	303 70.5%	127 29.5%	430 100%
Yes	24 44.4%	30 55.6%	54 100%

Total	327 67.6%	157 32.4%	484 100%

**Table 3:** Relation between symptomatic convergence insufficiency and symptomatic accommodative insufficiency.

Symptomatic convergence insufficiency (CI)	Symptomatic accommodative insufficiency (AI)		Total
	No	Yes	
No	383 84.4%	71 15.6%	454 100%
Yes	13 43.3%	17 56.7%	30 100%

Total	396 81.8%	88 18.2%	484 100%

**Table 4:** Relation between classic/clinical convergence insufficiency (3 clinical criteria met), accommodative insufficiency, and astigmatism magnitude (most astigmatic eye). Chi-square analyses compared each diagnostic category (row) to the reference category: students who did not meet the clinical criteria for either CI or AI (first row of data, in italic). No significant differences were observed (all *p* values > 0.10).

Category	Astigmatism magnitude, most astigmatic eye (*n*, %)			Total
	<1.00 D *N* = 212	1.00 to <3.00 D *N* = 126	≥3.00 D *N* = 146	
*Reference: no clinical CI or AI*	*135* *44.6%*	*80* *26.4%*	*88* *29.0%*	*303* *100%*
Clinical CI	24 44.4%	14 25.9%	16 29.6%	54 100%
Clinical AI	70 44.6%	39 24.8%	48 30.6%	157 100%
Clinical CI without AI	7 29.2%	7 29.2%	10 41.2%	24 100%
Clinical AI without CI	53 41.7%	32 25.2%	42 33.1%	127 100%
Clinical AI and CI	17 56.7%	7 23.3%	6 20.0%	30 100%
Symptomatic CI	17 56.7%	7 23.3%	6 20.0%	30 100%
Symptomatic AI	46 52.3%	18 20.5%	24 27.3%	88 100%
Symptomatic CI without AI	5 38.5%	4 30.8%	4 30.8%	13 100%
Symptomatic AI without CI	34 47.9%	15 21.1%	22 31.0%	71 100%
Symptomatic AI and CI	12 70.6%	3 17.6%	2 11.8%	17 100%
